# Conditions Optimizing and Application of Laccase-mediator System (LMS) for the Laccase-catalyzed Pesticide Degradation

**DOI:** 10.1038/srep35787

**Published:** 2016-10-24

**Authors:** Xiaoting Jin, Xiangyang Yu, Guangyan Zhu, Zuntao Zheng, Fayun Feng, Zhiyong Zhang

**Affiliations:** 1 Key Laboratory of Food Quality and Safety of Jiangsu Province/State Key Laboratory Breeding Base/Key Laboratory of Control Technology and Standard for Agro-product Safety and Quality, Ministry of Agriculture of P. R. China, Nanjing, 210014, China; 2 Institute for the Control of Agrochemicals, Ministry of Agriculture of P. R. China, Beijing, 100125, China

## Abstract

A high capacity of laccase from *Trametes versicolor* capable of degrading pesticides has been revealed. The conditions for degrading of five selected pesticides including chlorpyrifos, chlorothalonil, pyrimethanil, atrazine and isoproturon with the purified laccases from *Trametes versicolor* were optimized. The results showed that the optimum conditions for the highest activity were pH at 5.0 and temperature at 25 °C. The best mediators were violuric acid for pyrimethanil and isoproturon, vanillin for chlorpyrifos, and acetosyringone and HBT for chlorothalonil and atrazine, respectively. The laccase was found to be stable at a pH range from 5.0 to 7.0 and temperature from 25 to 30 °C. It was observed that each pesticide required a different laccase mediator concentration typically between 4.0–6.0 mmol/L. In the experiment, the degradation rates of pyrimethanil and isoproturon were significantly faster than those of chlorpyrifos, chlorothalonil and atrazine. For example, it was observed that pyrimethanil and isoproturon degraded up to nearly 100% after 24 hours while the other three pesticides just reached up 90% of degradation after 8 days of incubation.

Pesticides are important chemicals and widely used in modern agriculture to control pests and improve the commodity yield and quality. Up to 80% of crop yields could have had been lost without the proper uses of pesticides^1^,^2^. However, indiscriminating and excessive use of pesticides could lead to contamination of the produce and pollution of the environment^3^. In the literature, many reports have shown that pesticide exposures associated to adverse health effects such as depression, memory disorders, respiratory problems, dermal damage, neurological deficiency, miscarriages, birth deformities and cancer^4^. Recently, pesticide residue in/on food has become a major concern for food safety worldwide.

Bioremediation is an innovative technology that has the great potential to alleviate such problems by decreasing the pesticide residues in food and reducing the pollutant levels in the environment. Therefore, this work focuses on the microorganisms of naturally occurring microbial consortia for pesticide degradation^5^.

Laccase belongs to the family of blue copper-containing polyphenol oxidase. It was first described in latex obtained from Japanese tree *Rhus vernicifera* by Yoshida^6^. Laccases are enzymes widely produced by plants, fungi, bacteria, and some insects. The structures of laccases contain four copper ions which can catalyze the oxidation reaction with the presence of oxygen, directly leading to the decomposition of various phenolic compounds, such as phenolic dye, substituted phenol, chlorophenol and sulfur phenol, bisphenol A, and some aromatic amines, etc. Because of its catalytic oxidation by oxygen in the air, it is called “green catalyst”. It appeared that laccases only could degrade structure unit of phenol due to the low oxidation reduction potential of laccase (0.5 to 0.8 V)^7^. Bourbonnais reported that the laccase oxidized a non-phenol type with transferring electronic media^8^. It was generally considered that a laccase mediator system (LMS) contained laccase catalytic substances biologically interacting with the oxygen and the mediator. LMS was originally used in the biodegradation of lignin. Over the past few decades, the applications of LMS in the fields of biobleaching^9^,^10^, modification of pulp^11^, and paper mill effluent^12^ have been of a great concern. It was applied to decolorize dyes, bleach textile, treat wastewater^13^,^14^,^15^, and degrade of other refractory substances such as polycyclic aromatic hydrocarbons^16^,^17^, and hormone-like chemicals^18^,^19^.

In order to better understand the pesticide degradation by laccases, this research group optimized the conditions of laccase from white-rot fungus *Trametes versicolor* for degrading five selected pesticide, including chlorpyrifos, chlorothalonil, pyrimethanil, atrazine, and isoproturon, with the presence of different mediators at different pH, temperature and mediator concentrations.

## Results

### Effects of pH and temperature on the laccase activity

The results of relative activity are given in Tables 1 and 2. Overall, the relative activities of laccases decrease as the incubation day increases. As can be seen, the effects are different with incubation temperature (Table 1) and pH (Table 2). In Table 1, the relative laccase activities on day 8 were 70.4%, 74.3%, 71.6%, 40.5%, and 11.5% for the incubation temperature of 25, 30, 20, 35, and 40 °C, respectively. Thus, the optimum temperature is 25 °C (with no significant change at 20–30 °C, 8 days incubation). In Table 2, the relative laccase activities on day 8 were 71.6%, 65.7%, 60.3%, 27.7%, 2.7%, and 0% for the pH of 5.0, 6.0, 7.0, 4.0, 3.0, and 2.0, respectively. It should be noted that, after 24 hours incubation, the relative laccase activities decreased to 6.8% and 14.2% when pH at 2.0 and 3.0, respectively (Table 2). From 24 hours to day 8, in Table 2, the relative laccase activities were observed to be relatively stable, i.e., no significant changes within the experimental error. In contrast, in Table 1, the relative laccase activities seemed to have clear decreasing trends with incubation days. For example, at 40 °C, the relative activity of laccase was 100%, 71%, 42%, 29%, 16% and 14% on days 0, 1, 2, 4, 6, and 8, respectively.

### Roles of the mediators in the process of pesticide degradation

**Figure 1:**
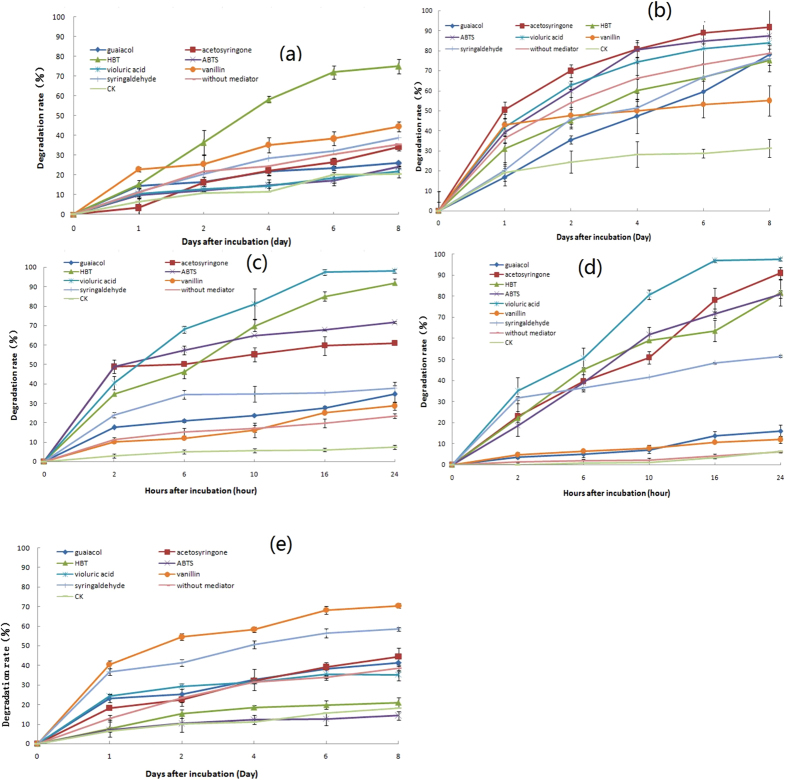
Effects of laccase mediator system (LMS) on pesticide degradation (pH 5, temperature 25 °C) (**a**): atrazine; (**b**): chlorothalonil; (**c**): isoproturon; (**d**): pyrimethanil; (**e**): chlorpyrifos).

White-rot fungi with an appropriate mediator are able to degrade the pesticides. The results are presented in Fig. 1. Data showed that the laccase extracted from *Trametes versicotor* with an appropriate mediator promoted the degradations of five selected pesticides including atrazine, chlorothalonil, isoproturon, pyrimethanil and chlorpyrifos. Comparing to the treatments of CK and without a mediator, the optimizing mediators were HBT for pesticide atrazine. Its degradation rate was up to 75.0% with HBT as a mediator. But with other mediators, the rates were found to be ranging from 21.7% to 38.9%. For isoproturon and pyrimethanil, the best mediators were found to be acetosyringone, ABTS, HBT and violuric acid. Syringaldehyde and vanillin were the best mediators for chlorpyrifos only. As can be seen in Fig. 1, the mediators play important roles in the degradation of the pesticides by laccase catalysis except for chlorothalonil. The degradation of isoproturon and pyrimethanil reached above 60% in the presence of a proper mediator 6 and 10 h incubation, respectively. However, it took 2 days for other selected pesticides to reach the similar degradation (60%). The data demonstrated that the degradation rates of isoproturon and pyrimethanil were higher than other three pesticidee. With violuric acid as a mediator, the decline rates of isoproturon and pyrimethanil were approximately 98% within 24 h. The degradation of atrazine, chlorothalonil, and chlorpyrifos ranged from 70.4% to 91.6% (8 days) with an appropriate mediator. However, without any mediator, the degradation rate of chlorothalonil was 78.6%. Similarly, for other four pesticides, the rates were from 6.1% to 38.9%.

### Optimization of pH, temperature and mediator concentration for pesticide degradation

**Figure 2:**
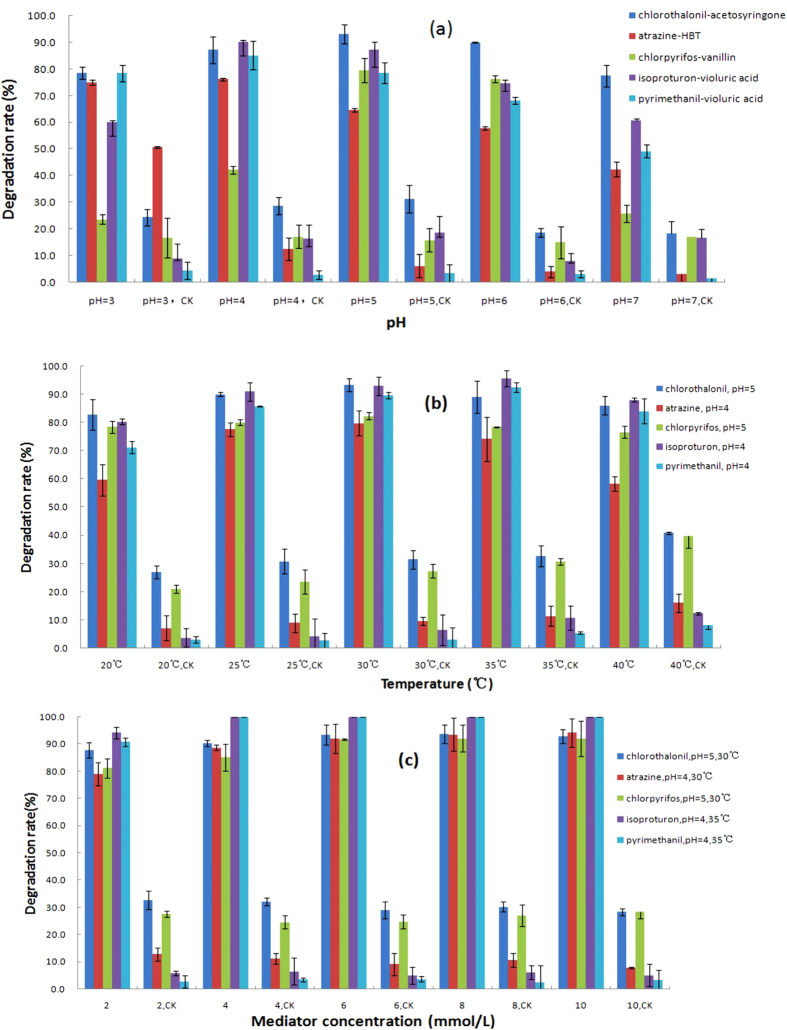
Effects of pH (**a**), temperature (**b**) and mediator concentration (**c**) on pesticide degradation (Incubation days: 8 days for chlorothalonil, atrazine and chlorpyrifos, 1 day for isoproturon and pyrimethanil).

In order to obtain the optimum conditions for pesticide residues in the biodegradation, pH of culture solution, incubation temperature and mediator concentration were studies. The pH values for laccase were determined by measuring the pesticide degradation rates in a citric acid-dibasic sodium phosphate buffer at varying pHs (pH 3–7). As can be seen in Fig. 2a, comparing to CK treatments, the pH optima were at 4.0 for chlorothalonil, isoproturon and pyrimethanil while for atrazine and chlorpyrifos, their optimum pHs were found to be pH 5.0. The best temperatures for the degradations of chlorothalonil, atrazine, chlorpyrifos and isoproturon, pyrimethanil demonstrated at 30 and 35 °C, respectively (Fig. 2b). The rates of pesticide degradation were found to be greater than 79% with the mediator concentrations ranging from 2.0 to 10.0 mmol/L. For isoproturon and pyrimethanil, the rates reached to nearly 100% with 4.0 mmol/L of mediator after 1 day treatment. The results suggested that the pesticide degradation rates increased with increasing the fortification of the mediator concentration until it reached the maximum, i.e., approximately 100% at 8.0 mmol/L (Fig. 2c).

## Discussion

Temperature, pH and mediator play important roles in laccase activity and its catalyzed pesticide degradation. In the literature, most of the fungal laccase optimum pH is 5.0 (form 4.0 to 6.0). For example, the pH optimum was at 5.0 for the laccase from the edible wild mushrooms, including *Albatrella dispansus*^20^, *Cantharellus cibarius*^21^. *Coriolus hirsutus*^22^, *Lentinula edodes*^23^ and *Tricholoma giganteum*^24^ and pH 4.0 for *Hericium erinaceum*^25^ and *Panus tigrinus*^26^. However, in terms of maximal activities, these reports indicated that the most of laccases required higher incubation temperatures in the previous work than the that for selected *Trametes versicolor* in this study. The laccases from *C. cibarius* and *H. erinaceum* which had an optimum temperature at 50 °C, while *C. hirsutus* and *R. lignosus* were at 45 °C and 40 °C, respectively. Laccase requires a temperature of 40 °C to exhibit its maximal activity. The high activity observed at 35–45 °C for *Rigidoporus lignosus* (38). As shown in Table 2, an optimum temperature of 25 °C was required for the laccase from *Trametes versicolor* to reach its maximal activity. When the temperature was at 20 and 30 °C, it also showed a good stability of laccase activity in this study.

The results demonstrated that the laccase from *Trametes versicolor* with an appropriate mediator could accelerate the degradations of five selected pesticides compared to the treatments of without a mediator. A number of studies reported the capacity of removal of pesticides in contaminated agricultural environment by LMS. Purified phenol oxidase (laccase) from the white rot fungus *Pleurotus ostreatus* (Po) together with the mediator of 2,2′-azinobis(3-ethylbenzthiazoline-6-sulfonate) (ABTS) indicated complete and rapid oxidative degradations of the nerve agents O-ethyl S-[N,N-diisopropylaminoethyl]methylphosphonothiolate (VX) and O-isobutyl S-[N,N-diethylaminoethyl]methylphosphonothiolate (RVX) and the organophosphorus insecticide analog diisopropyl-Amiton. A molar ratio of 1:20 for OP/ABTS and 0.05 M phosphate at pH 7.4 provided the highest degradation rate of VX and RVX^27^. The laccase from *Trametes versicolor* showed incomplete but effective degradation of VX, O-ethyl-S-[2-(diisopropylamino)ethyl] phenylphosphonothioate (PhX) and RVX in the presence of ABTS^28^. The results of twelve halogenated organic pesticide analyses in the presence of nine different mediators indicated that the acetosyringone and syringaldehyde appeared to be the best mediator^29^. The degradation rates of herbicide glyphosate 24 hours incubation were 40.9%, 62.8% and 90.1% by three kinds of mixture of laccase and ABTS, Mn^2+^, Tween 80 with laccase, respectively^3^. Fungicide cyprodinil did not show transformation when incubated alone with a laccase from *Trametes villosa*. But it was transformed to a significant extent, when a mediator was present^30^. In the literatures, the laccase-catalyzed pesticide degradations depended on selected pesticide physicochemical properties and different optimum of LMS. The pH optima for fungal laccases rely generally on the acidic region. The pH optima were reported that had a wide range showing from 2.7 to 7.5, and typically in the range of 3.5 to 6.0, depending on the different substrate^31^,^32^. Amitai *et al.* reported that the optimal pH for degradation of VX and RVX was 7.4 as compared to DiPr-Amiton that is degraded more rapidly at pH 8.0^27^. Stepanova *et al.* showed that the pH optimum of catechol oxidation by *Pleurotus oastreatus* 0432 laccase was 6.0^33^. The results demonstrated that the LMS with pH optima for chlorothalonil and chlorpyrifos were at 5.0, for atrazine, isoproturon and pyrimethanil were at 4.0, respectively.

Mediator concentration is also a factor in laccase-catalyzed pesticide degradation. The optimal molar ratio of ABTS/OP for VX and RVX degradation was 1:20, whereas, the rate of diPr-amiton degradation rate reached its peak at a molar ratio of 1:10^27^. The natural mediator syringaldehyde showed to be an efficient mediator and the highest pesticide transformation rates of recalcitrant halogenated pesticides were obtained with a mediator–substrate proportion of 5:1^29^. For all the selected pesticides in this study, the maximum activity was reached at a mediator concentration of 4.0 mmol/L, which was slightly higher than the values in the literature. But the initial concentrations of the selected pesticides in this study (20.0 mg/L for each) were significantly higher than those above-mentioned.

## Methods and Materials

### Instruments

Gas chromatography was an Agilent GC 7890 (Agilent Technologies, Santa Clara, CA, USA) equipped with a μ-ECD detector and an analytical column HP-5ms J&W Ultra Inert capillary column (30 m length × 0.25 mm I.D × 0.25 μm film thickness, Agilent Technologies, USA). High performance liquid chromatography (Agilent Technologies, USA) contained a 1200 SL HPLC system with an Agilent ZORBAX SB-C18 (2.1 × 150 mm, 5 μm) analytical column.

### Pesticide standards

Chlorpyrifos (purity: 99.9%), chlorothalonil (purity: 97.5%), pyrimethanil (purity: 97.5%), atrazine (purity: 99%), isoproturon (purity: 99%) were purchased from Dr. Ehrenstorfer Company (Germany).

### Reagents

Laccase purified from *Trametes versicolor* were purchased from Sigma (USA). Organic solvents, n-hexane, acetone, acetonitrile, methanol, sodium chloride and petroleum ether, were of analytical grades and purchased from Kermel Chemical Reagent Co., Ltd (Tianjin, China). Pesticide standard stock solutions (1000 mg/L) were prepared in acetone and stored in a refrigerator at 4 °C. HBT, ABTS, violuric acid, and acetosyringone were purchased from Oddo’s Biological Co., Ltd (Nanjing, China). Syringaldehyde and vanillin were purchased from Aladdin (Shanghai, China). Guaiacol was purchased from Chinese Medicine Group Chemical Reagent Co., Ltd. (Beijing, China).

### Measurement of laccase activity

Laccase activity was measured at 420 nm by generation of ABTS2^+^ radicals from the enzymatic oxidation of ABTS at 25 °C using a spectrophotometer. The assay mixture contained 200 μL of 0.5 mmol/L ABTS, 2700 μL of sodium acetate buffer (pH 4.5) and 100 μL of the enzyme-containing sample. One unit of laccase activity (U) was defined as the amount of enzyme that formed 1 μmol ABTS per min, using the extinction coefficient ε 420 nm of 36,000 /M/cm^34^.

### Influence of the pH and temperature on laccase stability

Laccase was added in citric acid-dibasic sodium phosphate buffers with the pH at 2.0, 3.0, 4.0, 5.0, 6.0 and 7.0. The incubation system were in tubes put in an incubator, and the temperature was controlled at 25 °C. Samples were collected at 1, 2, 4, 6 and 8 days intervals after laccase application for the detection of laccase activity. Laccase activity was controlled in 50 U/L of each treat at the beginning. Laccase was separately added to citric acid-dibasic sodium phosphate buffers with the pH at 5.0. The incubation system were in tubes put in the incubator, and the temperature was controlled at 20, 25, 30, 35, and 40 °C, separately. Samples were collected at 1, 2, 4, 6 and 8 days after Laccase application for the detection of laccase activity.

### Degradation of pesticides by laccase

Degradation experiment were carried out in 10 mL tubes in citric acid-dibasic sodium phosphate buffers containing 20.0 mg/L pesticides in the dark at 30 °C in the incubator. The enzyme activity was in the control of 0.05 U/mL by an ultraviolet spectrophotometer. Samples of atrazine, chlorothalonil, chlorpyrifos, and pyrimethanil, isoproturon were collected at 1, 2, 4, 6, 8 days and 0, 2, 6, 10, 16, 24 h after incubation, respectively. Then the residues were measured by HPLC or GC. All of the treatments including controls were in triplicates.

### Pesticide residues analysis

For chlorothalonil, pyrimethanil, chlorpyrifos and isoproturon, 2.0 mL of the buffer solution was added into a 10 mL glass tube, aloowed by adding 2.0 mL of petroleum ether to the extract. The mixture was vortexed for 2 min and wait for the layer separation. The supernatant fluid (petroleum ether, organic phase) was transferred into a clean glass tube. Repeat the above steps 2 additional times. Then the supernatants were combined. Three (3.00) mL of the supernatant was transferred into another tube and dried under a stream of nitrogen (40 °C). The pesticide residues were dissolved in 1 mL of appropriate organic solvent* and filtered through a 0.22 μm nylon filter into an autosampler vial. (*The residue of chlorpyrifos was dissolved in n-hexane for GC analysis, chlorothalonil and pyrimethanil were dissolved in methanol for HPLC analysis, and isoproturon were dissolved in acetonitrile for HPLC analysis. For atrazine, it was diluted using acetonitrile for HPLC analysis just after filtered using a 0.22 μm nylon filter without additional extraction or cleanup steps).

### GC analysis

#### Chlorpyrifos

The conditions for the analysis were: detector temperature 300 °C, injector temperature 250 °C, oven temperature program starting at 120 °C, 13.5 min at 120–270 °C (ramp 20 °C/min), carrier gas, N_2_ at 1 mL/min, injection volume 1.0 μL, and splitless mode. A linear calibration curve was used and the calibration range was 0.01–5 mg/L. The retention time of chlorpyrifos was approximately 5.8 min.

#### HCLP analysis. Atrazine

The mobile phase containing acetonitrile/water (75:25, v/v) was delivered at a flow rate of 0.8 mL/min. The injection volume was 20 μL and detection wavelength was 222 nm. The calibration curve was obtained using peak area versus concentrations and the calibration range was 0.01–5 mg/L. The retention time of atrazine was approximately 4.2 min.

#### Isoproturon

The mobile phase containing acetonitrile/water (70:30, v/v) was delivered at a flow rate of 0.3 mL/min. The injection volume was 10 μL and detection wavelength was 245 nm. The calibration range was 0.01–5 mg/L. The retention time of isoproturon was approximately 10.4 min.

#### Chlorothalonil

The mobile phase contains methanol/water (75:25, v/v) flow rate 0.7 mL/min, injection volume 20 μL and detection wavelength 240 nm. The calibration range was 0.01–5 mg/L. The retention time of chlorothalonil was approximately 9.7 min.

#### Pyrimethanil

The mobile phase contained methanol/water (75:25, v/v), flow rate 1.0 mL/min, injection volume 20 μL and detection wavelength 270 nm. The calibration range was 0.01–5 mg/L. The retention time of pyrimethanil was approximately 4.6 min.

## Additional Information

**How to cite this article**: Jin, X. *et al.* Conditions Optimizing and Application of Laccase-mediator System (LMS) for the Laccase-catalyzed Pesticide Degradation. *Sci. Rep.* **6**, 35787; doi: 10.1038/srep35787 (2016).

## Figures and Tables

**Table 1:** Effects of temperature on the relative activity (%) of laccase (pH at 5.0).

Temperature (°C)	Days after incubation (day)					
	0	1	2	4	6	8
20	100 ± 2.4	85.5 ± 4.2	82.1 ± 7.4	80.4 ± 3.6	74.3 ± 5.4	70.4 ± 6.3
25	100 ± 5.4	98.0 ± 5.3	96.6 ± 3.0	89.9 ± 3.6	81.1 ± 7.4	74.3 ± 8.4
30	100 ± 6.4	96.6 ± 2.4	93.2 ± 5.3	86.5 ± 7.3	81.1 ± 4.3	71.6 ± 4.6
35	100 ± 3.4	77.7 ± 7.5	73.0 ± 2.5	56.1 ± 4.8	45.9 ± 3.7	40.5 ± 4.6
40	100 ± 7.5	71.6 ± 3.3	41.9 ± 2.5	28.4 ± 3.2	13.5 ± 2.1	11.5 ± 3.1

**Table 2:** Effects of pH on the relative activity (%) of laccase (at 25 °C).

pH	Days after incubation (day)					
	0	1	2	4	6	8
2.0	100 ± 4.0	6.8 ± 0.1	4.1 ± 0.0	0	0	0
3.0	100 ± 6.4	14.2 ± 1.2	10.8 ± 0.4	7.4 ± 0.1	3.4 ± 0.2	2.7 ± 0.2
4.0	100 ± 8.4	50.0 ± 4.5	41.9 ± 4.2	33.1 ± 2.5	29.3 ± 4.2	27.7 ± 3.2
5.0	100 ± 3.4	96.6 ± 3.5	93.2 ± 7.5	81.1 ± 2.4	80.1 ± 4.3	71.6 ± 4.3
6.0	100 ± 9.5	75.7 ± 11.3	75.0 ± 7.5	72.4 ± 7.4	70.7 ± 7.2	65.7 ± 6.4
7.0	100 ± 5.3	73.6 ± 3.3	72.3 ± 6.4	70.7 ± 3.6	63.6 ± 4.4	60.3 ± 4.8
